# Wave of change: assessing surf therapy’s psychological and physiological benefits for military veterans using wearable technology

**DOI:** 10.3389/fpsyg.2025.1613418

**Published:** 2025-09-16

**Authors:** Jonathan E. Ossie

**Affiliations:** Department of Marketing, Fowler College of Business, San Diego State University, San Diego, CA, United States

**Keywords:** flow, surf therapy, alternative therapy, technology, mental health

## Abstract

Mental health issues, particularly among military veterans, are a growing concern. Veterans disproportionately experience anxiety, depression, and PTSD, often exacerbated by combat-related stressors. While pharmacological treatments yield mixed outcomes, interest in nonpharmacological interventions like surf therapy is increasing; however, empirical evidence remains limited. This study evaluated the impact of a weeklong surf therapy program conducted by Operation Surf on 41 veterans with PTSD using a mixed-methods approach: self-reported surveys and wearable technology (Whoop bands) measuring physiological data. Psychological outcomes were assessed using GAD-7, PHQ-8, and PCL-5; physiological outcomes included heart rate variability (HRV) and sleep quality. Results revealed: A 59% reduction in general anxiety (GAD-7) immediately after the event (mean decrease = 7.38, *p* < 0.01), and 30% reduction at 30 days (*p* < 0.05). A 44% reduction in depression (PHQ-8) immediately after the event (mean decrease = 4.94, *p* < 0.01), and 30% reduction at 30 days (*p* < 0.01). A 38% reduction in PTSD symptoms (PCL-5) immediately post-event (mean decrease = 16.95, *p* < 0.01), and 35% reduction at 30 days (*p* < 0.01). Wearable technology indicated a statistically significant decrease in HRV during the intervention (*p* < 0.05), and sex-specific changes in deep and REM sleep, particularly in female participants. These findings demonstrate statistically and clinically meaningful improvements in psychological functioning and highlight the utility of wearable technology in evaluating complementary mental health interventions. While cost and stigma were not directly measured, the results support further investigation into the broader applicability and accessibility of surf therapy for veterans with PTSD.

## Introduction

As the COVID-19 pandemic extended into its third year, the mental health crisis deepened, with a significant rise in conditions such as anxiety, depression, substance abuse, and suicidal ideation, exacerbating the pre-existing mental health landscape. Pre-pandemic estimates indicated over 51.5 million U.S. adults suffered from mental illness, a situation likely worsened by the pandemic’s strain (Merikangas et al., 2010; Substance Abuse and Mental Health Services Administration, 2014; Czeisler et al., 2020). Trauma, whether from international conflicts, domestic violence, or substance abuse within families, has long been a source of mental and physical health challenges, leaving lasting impacts on individuals’ psyches and overall health (van der Kolk, 2014; Black et al., 2011).

Particularly vulnerable to these conditions are military veterans, who face a heightened risk of posttraumatic stress disorder (PTSD) and related mental health issues due to their experiences in combat and difficulty readjusting to civilian life (Benninger et al., 2020; Crawford, 2016; Milliken et al., 2007; Oman and Bormann, 2015; Rogers et al., 2014). PTSD manifests through a range of symptoms, including reexperience, avoidance, negativity, and hyperarousal, significantly affecting veterans’ interpersonal relationships, self-efficacy, mental, and physical functioning, diminishing psychological resilience, leading to increased risks of suicide and other severe consequences (National Institute of Mental Health, 2020; Crawford, 2016; Jakupcak et al., 2007; Milliken et al., 2007; Oman and Bormann, 2015; Pietrzak et al., 2009; Vasterling et al., 2009).

The sheer volume and scope of mental health issues, exhibited by the number of affected as well as through the concomitant economic, financial, and relational costs and strains, necessitates further investigation into various treatment modalities. Although several pharmacological approaches have been employed to combat this epidemic, their efficacy is mixed at best, which has led to several novel, nonpharmacological approaches. Furthermore, some individuals also may not prefer, have access to, or benefit from, traditional therapies (Walter et al., 2019). Across military studies, one of the most frequently reported barriers to help-seeking mental health problems is concerns about stigma (Sharp et al., 2015).

Amid these challenges, complementary and alternative medicine (CAM) options, including nature-based therapies like surf therapy, have emerged as promising avenues for treating PTSD and improving mental health outcomes (Thompson Coon et al., 2011; Barton and Pretty, 2010). These therapies, leveraging the healing potential of the natural environment and physical activity, have shown significant promise in reducing symptoms of PTSD, depression, and stress, and improving functional impairment, self-efficacy, and leisure satisfaction among veterans (Bennett et al., 2017; Wheeler et al., 2020; Walter et al., 2019). It typically takes place in group settings and incorporates principles of physical activity, nature exposure, social connection, and therapeutic facilitation (Benninger et al., 2020).

One nonpharmacological approach is Operation Surf, a nonprofit organization that provides nature-based programs advocating the restorative power of the ocean and surfing. Previous quantitative research on ocean therapy using data gathered from survey instruments have indicated a significantly positive impact on veterans, showing a reduction in PTSD symptoms, reduced depression, and an increase in self-efficacy among veterans with PTSD in the United States (Crawford, 2016).

These positive impacts on veterans through programs such as Operation Surf could prove to be a viable, nature-based intervention for those struggling with the transition to civilian life and its associated challenges (Crawford, 2016; Rogers et al., 2014).

Surf therapy has garnered attention for its potential to aid veterans in overcoming trauma, boosting confidence, and fostering a sense of purpose through the therapeutic power of the ocean. Although surf programs for military veterans exist, data evaluating such programs are limited, and additional research is needed to further understanding.

This growing interest in CAM and nature-based therapies coincides with advancements in wearable technology, which offers new opportunities for monitoring and improving health outcomes. Wearables can unobtrusively collect physiological biomarker data, providing insights into fitness, sleep, and recovery, and offering a novel approach to managing PTSD and other mental health conditions (Bayoumy et al., 2021; Castaneda et al., 2018; Wu and Luo, 2019). Wearable technology allows for the passive, continuous collection of physiological biomarkers in real-world settings, offering a complementary lens to self-reported psychological outcomes. Two markers of particular relevance are heart rate variability (HRV) and sleep architecture, including REM and deep sleep. HRV is a well-established indicator of autonomic nervous system function and has been inversely associated with stress, PTSD severity, and depression in both clinical and military populations (Dennis et al., 2016; Minassian et al., 2015). Lower HRV has been linked to increased emotional dysregulation and diminished resilience. Similarly, disrupted sleep—especially reduced REM and deep sleep—has been documented as a core symptom and outcome predictor for PTSD, anxiety, and depression (Germain, 2013). Wearables provide a unique opportunity to track these markers non-invasively across time and intervention settings, making them well-suited for evaluating the impact of surf therapy interventions.

The purpose of this research study was to collect data for analysis and insight using new technologies to measure therapy effectiveness. Specifically, the purpose of this study was to understand how Operation Surf’s weeklong ocean surf therapy program impacted psychological, physical, and functional outcomes for veterans seeking mental health treatments for symptoms of PTSD. Thus, survey data was used to measure changes in depression, anxiety, and PTSD—together with physiological data generated from Whoop bands—to produce a more robust set of programmatic efficacy inferences for six cohorts of military veterans who participated in Operation Surf from May 2021 through May 2022.

This research addresses the knowledge gap in surf therapy studies, characterized by scarce empirical evidence and a limited range of methodologies and measurements.

Utilizing wearable technology, this study introduces an innovative approach to collecting objective physiological data in outdoor environments, previously unobtainable. These data provide a complementary lens to self-reported symptoms, enriching our understanding of the therapy’s impact on veterans’ health by capturing both subjective experiences and physiological correlates of change. This integration of utilizing technology collecting physiological data with therapeutic interventions addresses some limitations of previous research, while also allowing for a more comprehensive examination of the range of outcomes that may be impacted by engaging in outdoor physical activity (Callegaro et al., 2014). This research and wearable technology presents a promising pathway for enhancing mental health care, offering hope for more effective, personalized treatment options for PTSD and other mental health challenges.

The specific aim of this research project was to evaluate the effects of participating in Operation Surf’s weeklong surf therapy program. The objectives were to help provide evidence and build on the existing knowledge base, increase the scientific rigor of the body of research, generate additional data for analysis, inform public policy, and create evidence-based data to further understand and improve care.

This research addresses a critical gap in surf therapy literature, which has thus far lacked objective physiological data and longitudinal evaluation. By integrating wearable technology into the study of Operation Surf—a weeklong surf therapy program for military veterans—this project explores the potential for nature-based, group-oriented interventions to influence both psychological and physiological outcomes. The aim of the present study was to evaluate changes in PTSD, anxiety, and depression symptoms, as well as changes, if any, in biometric indicators such as sleep quality and heart rate variability, before, during, and after participation in the program. This work contributes to a growing body of research on complementary mental health treatments by combining real-world intervention data with emerging digital health tools.

## Methods

### Participants

Participants (*n* = 41) for this study were recruited using a nonprobability sampling strategy, as they were all attendees of Operation Surf weeklong events. In nonprobability sampling, study participants are selected based on availability, accessibility, convenience, and the fulfillment of the inclusion criteria for the study (Callegaro et al., 2014). All participants in Operation Surf weeklong surf events between May 2021 and May 2022 were eligible for study participation. To qualify, individuals were required to:

- Be an Active-Duty service member, Active Reservist, or Honorably Discharged Veteran of the U.S. Armed Forces;
- Have a service-connected injury, either visible (e.g., physical trauma) or invisible (e.g., psychological or neurological condition);
- Have served in the Post-9/11 era with one or more combat deployments;
- Complete the full application process, including a written essay meeting minimum length and content requirements; and
- Be available to attend the entire duration of the structured week-long surf therapy program (Operation Surf, 2024).

All participants who met these inclusion criteria and were available during the study period were invited to participate. Participation in the study was voluntary and they could withdraw from the assessments or program at any time without explanation.

While this study recorded participant sex, age range and combat deployment history, other socio-demographic and clinical background variables—including education level, employment status, disability rating, substance use, PTSD treatment history, and medication status—were not systematically collected. PTSD diagnosis was self-reported by participants during the Operation Surf application process and was not independently verified by the research team.

### Demographics

Of the 41 military veteran participants, 66% (*n* = 27) identified as male and 34% (*n* = 14) as female. The age range of participants was 28–56 years at their time of participation in the program, with the mean age of 41.6 (SD = 8.36). All participants were military veterans (Table 1).

**Table 1:** Participant demographics and military characteristics (*N* = 41).

Variable	*n*	%
Age mean (years)	41.60	
Age (standard deviation)	8.36	
Age range (years)	28–56	
Sex		
Male	27	66%
Female	14	34%
Combat deployment	41	100% (all participants)
PTSD diagnosis (self-report)	41	100% (inclusion criterion)

PTSD, posttraumatic stress disorder. Additional demographic or clinical data (e.g., education, occupation, treatment history) were not collected in this study and are acknowledged as a limitation.

### Program

The ocean surf therapy program was provided by Operation Surf, a nonprofit that delivers results-driven, nature-based programs advocating the restorative power of the ocean and surfing as a form of wellness for injured bodies, minds, and souls (Kroenke et al., 2009). The weeklong surf events took place near Avila Beach, CA, and Santa Cruz, CA.

Each Operation Surf program lasted 6 days, consisting of two travel days and four full days of adaptive surfing and structured wellness activities. Events were built around twice-daily surf instruction (morning and afternoon sessions of 2–3 h each), communal meals, and additional holistic components. Surfing sessions were moderately to vigorously strenuous, depending on ocean conditions and participant fitness. Instructors worked 1:1 or 1:2 with participants, offering physical and emotional support as needed. Participants wore wetsuits and used adaptive equipment to accommodate injuries, mobility limitations, or psychological concerns.

Mornings began with guided yoga and stretching sessions (~30 min) designed to enhance physical readiness and promote mindfulness through breathwork. All meals were eaten together in communal settings to encourage social bonding. A ceremonial opening blessing was conducted by members of the local Chumash tribe, connecting participants with the cultural and spiritual dimensions of the coastal environment. Each evening concluded with a “recap reel”—a structured group reflection during which participants viewed photos or videos from the day and shared insights or emotional responses, fostering connection, vulnerability, and therapeutic peer support.

While no formal psychotherapy was conducted, informal therapeutic dialogue occurred frequently among peers and instructors. The experience emphasized community building, self-efficacy, and emotional openness. Many participants shared stories related to trauma, service, and healing.

The study included six cohorts of military participants who took part in separate Operation Surf events between May 2021 and May 2022. All events followed a standardized curriculum, including daily surf instruction, yoga, communal meals, and structured reflection. Surf sessions were held at either Avila Beach or Santa Cruz, CA, with consistent timing and procedures. While instructor composition varied slightly, all lead staff were Operation Surf-trained and followed detailed safety and engagement protocols to ensure comparability across cohorts.

To support ongoing community and reinforcement of therapeutic benefits, participants were invited to join a private GroupMe text chat following the event. These chats were peer-led but occasionally supported by staff. In addition, optional weekly Zoom meetings were made available to program alumni as a form of virtual check-in and extended peer support. Participation in these follow-ups was voluntary but widely utilized, particularly by those seeking continued connection and accountability.

### Procedure

Participants were assessed at three timepoints: prior to the program (Time Point 1), immediately following the program (Time Point 2), and 30 days after completion (Time Point 3). At each point, data were collected using both self-report surveys and physiological measures to evaluate psychological symptoms and stress-related biomarkers.

Time Point 1 assessments (surveys and wearable data collection) were completed within 14 days prior to the start of the program, with most participants completing surveys within 5–10 days of arrival. The exact timing varied slightly due to travel logistics and participant availability, but all baseline assessments were completed before any exposure to the intervention. WHOOP bands were worn continuously during this pre-event window, providing 7 to 14 days of baseline physiological data depending on participant compliance and device access.

The three validated psychological instruments used in this study were the Generalized Anxiety Disorder-7 (GAD-7) to assess anxiety, the Patient Health Questionnaire-8 (PHQ-8) to assess depression, and the PTSD Checklist for DSM-5 (PCL-5) to assess PTSD symptoms. Concurrently, participants wore WHOOP biometric bands, which recorded physiological data including heart rate variability (HRV), REM sleep, and deep sleep. Participants were asked to wear the bands for up to 14 days prior to the program, continuously during the weeklong event, and for 30 days post-event to allow longitudinal comparison.

The GAD-7 and PHQ-8 assessed symptoms over the past 2 weeks, and the PCL-5 assessed symptoms over the past month. Time Point 1 assessments were completed up to 14 days prior to the start of the program, Time Point 2 assessments were completed immediately after the six-day program, and Time Point 3 assessments were completed 30 days after the program. This allowed for clear non-overlapping recall periods for GAD-7 and PHQ-8 across all timepoints. For the PCL-5, the Time Point 3 assessment reflects a full post-intervention symptom window. However, the Time Point 2 PCL-5 assessment may include some symptoms recall from before or during the intervention, and this is noted as a limitation in interpreting immediate post-program PTSD scores.

All data were collected using the Qualtrics platform for surveys and WHOOP’s secure data export for biometrics. Follow-up contact was indeed part of our data collection protocol to maximize survey completion. Specifically, participants received automated email reminders at 24 h, 3 days, and 7 days post-survey distribution. The study protocol was approved by the Institutional Review Board (IRB), and all participants provided informed consent. This multimodal data collection approach allowed for both subjective and objective evaluation of the Operation Surf program’s short-term and sustained impact.

**Figure 1:**
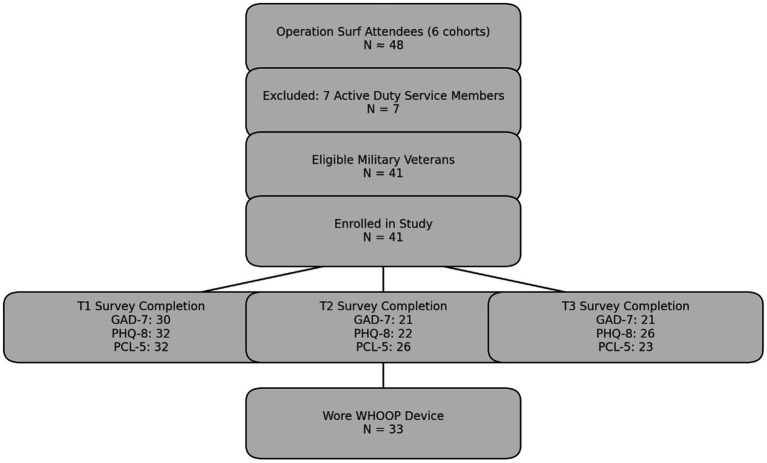
Participant flow diagram for surf therapy feasibility study.

Survey completion rates varied across time points and measures but remained consistent with expectations for feasibility studies involving high-risk populations such as military veterans with PTSD. For depression, the Patient Health Questionnaire-8 (PHQ-8) was completed by 32 participants (78%) at Time Point 1 (pre-intervention), 22 participants (54%) at Time Point 2 (immediately post-intervention), and 26 participants (63%) at Time Point 3 (30-day follow-up) (Figure 1). To assess anxiety, the Generalized Anxiety Disorder-7 (GAD-7) was completed by 30 participants (73%) at Time Point 1, and by 21 participants (51%) at both Time Points 2 and 3. For PTSD symptoms, the PTSD Checklist for DSM-5 (PCL-5) was completed by 32 participants (78%) at Time Point 1, 26 participants (63%) at Time Point 2, and 23 participants (56%) at Time Point 3. Physiological data were obtained from 33 participants (80%), limited by equipment unavailability due to a supply chain delay in November 2021 (8 participants, 20%). This variation reflects real-world constraints of longitudinal research in field-based, non-clinical environments. These completion and retention trends are consistent with feasibility research norms for high-burden or trauma-exposed populations, where follow-up rates between 50–70% are common due to complex psychosocial factors (Eldridge et al., 2016; Robinson et al., 2007). While the primary clinical outcomes in this study were patient-reported symptoms of PTSD, anxiety, and depression, physiological data collected from WHOOP wearables were used to supplement and contextualize these outcomes. Specifically, HRV, REM sleep, and deep sleep served as objective markers of stress regulation and recovery, intended to evaluate potential physiological correlations of psychological change. These biometrics were not used as primary clinical endpoints, but rather as exploratory measures to assess the feasibility and utility of integrating wearable technology in surf therapy research.

All study procedures were reviewed and approved by the Institutional Review Board (IRB) at Pearl, under protocol number (IRB#2023-0207). All participants provided informed consent prior to data collection, and participation was entirely voluntary. Participants could withdraw from the study at any point without penalty.

### Measures

#### Anxiety

Anxiety severity was assessed across three points in time, from preprogram, through event and for 30 days following event, using the Generalized Anxiety Disorders 7-item measures (GAD-7). The seven symptoms are rated on a scale from one to three and summed for a total score, with higher total scores indicating greater severity of anxiety. The 7-item anxiety scale has strong reliability (*α* = 0.92) and strong criterion, construct, factorial, and procedural validity (Spitzer et al., 2006).

### Depression

Depression was assessed using the Patient Health Questionnaire-8 (PHQ-8), a validated self-report instrument widely used to measure the severity of depressive symptoms in clinical and research settings (Kroenke et al., 2009). PHQ-8 consists of eight items that correspond to the diagnostic criteria for major depressive disorder outlined in the DSM-IV. Respondents are asked to rate how often they have experienced each symptom over the past 2 weeks on a scale from 0 (not at all) to 3 (nearly every day), with total scores ranging from 0 to 24. Higher scores indicate greater severity of depressive symptoms. The PHQ-8 has demonstrated strong internal consistency (Cronbach’s *α* = 0.86–0.89) and is commonly used in both clinical and research contexts to track changes in depression severity over time (Kroenke et al., 2001; Kroenke and Spitzer, 2002).

### PTSD symptoms

PTSD symptoms were assessed using the PTSD Checklist for DSM-5 (PCL-5), a 20-item self-report measure that evaluates the presence and severity of PTSD symptoms based on the 2013 published DSM-5 criteria. Respondents are asked to rate how much they have been bothered by each symptom in the past month on a scale from 0 (not at all) to 4 (extremely), resulting in total scores ranging from 0 to 80. Higher scores indicate greater symptom severity, and a cutoff score of 31 was used to suggest probable PTSD (Spitzer et al., 2006). The PCL-5 has demonstrated strong internal consistency (Cronbach’s *α* = 0.94) and test–retest reliability, making it a widely used and validated tool for both clinical assessment and research purposes (Wortmann et al., 2016; Weathers et al., 2014). It allows for the measurement of overall PTSD severity as well as symptom clusters related to reexperiencing, avoidance, negative alterations in mood and cognition, and hyperarousal (National Center for PTSD, 2023). The PCL-5 is sensitive to treatment-related changes, with a 5–10-point change typically referred to as reliable, and a reduction of 10 to 20 points is considered clinically meaningful improvement in PTSD symptoms, indicating a significant reduction in symptom severity that reflects a noticeable impact on the individual’s functioning or quality of life.

### Physiological biomarkers

WHOOP wearable devices collect a wide range of physiological data, including sleep architecture, heart rate metrics, strain/load, and respiratory patterns. For this study, we selected heart rate variability (HRV), REM sleep, and deep sleep as primary indicators due to their established relevance in mental health research. HRV is a validated marker of autonomic nervous system functioning and stress response (Berryhill et al., 2020), while REM and deep sleep are critical for emotional regulation and physical restoration, respectively (Germain, 2013; Minkel et al., 2012). These variables have been repeatedly linked to symptom severity and treatment outcomes in PTSD, anxiety, and depression. Other available measures—such as strain, calories burned, or sleep onset latency—were excluded due to limited or indirect relationships with psychological well-being.

HRV, a key indicator of autonomic nervous system function and overall stress, was measured continuously throughout the study. HRV reflects the variation in time between heartbeats, with higher variability typically associated with greater parasympathetic (rest and digest) activity and better physiological resilience (Shaffer and Ginsberg, 2017). In this study, HRV was reported in milliseconds (ms) as a single nightly value calculated by the WHOOP device using the Root Mean Square of Successive Differences (RMSSD), a time-domain measure that specifically reflects parasympathetic (vagal) tone. These values were calculated during deep sleep, a period chosen by WHOOP for its low noise and stable physiological conditions. While WHOOP uses internal algorithms to correct for motion artifacts and signal noise, no additional post-processing or raw data filtering was performed by the research team.

Participants were instructed to wear the device continuously throughout the study period, including during sleep and waking hours. However, detailed backend wear-time logs were not utilized, and we did not quantify exact wear-time or evaluate missing data on a per-participant basis in this feasibility study. Nights with incomplete sleep data or missing HRV values were excluded from analysis. We acknowledge this as a limitation and recommend that future research utilize direct access to wear-time metrics and data quality indicators to better evaluate HRV signal integrity and compliance.

Sleep metrics, including total sleep duration, deep sleep, and REM sleep, were also tracked using the wearable devices. Deep sleep is associated with physical restoration and recovery, while REM sleep is critical for cognitive functions and emotional regulation. Sleep data were automatically recorded, capturing key variables such as total hours slept and time spent in different sleep stages. These data offer objective measurements of sleep quality, which are particularly relevant for individuals with PTSD, as sleep disturbances are common in this population.

REM and deep sleep were included in the analysis as exploratory variables due to their theoretical relevance to trauma recovery and emotional regulation. While WHOOP does not provide the same level of precision as polysomnography for sleep-stage classification, its use has been validated for estimating total sleep time and sleep onset with reasonable accuracy in free-living conditions (Berryhill et al., 2020). Its ability to unobtrusively collect sleep-stage estimates over multiple nights in naturalistic settings made it well-suited for this feasibility study. These stage estimates were used to examine general trends across participants, not as definitive clinical endpoints, and should be interpreted accordingly. Future studies may benefit from combining wearable-derived sleep metrics with clinical sleep evaluations.

Wearable technology allowed for the unobtrusive collection of continuous physiological data in real-world settings, minimizing participant burden and providing a more comprehensive understanding of how surf therapy interventions influenced both physiological stress and sleep patterns. These biomarkers complemented the self-reported psychological data, offering a holistic view of the program’s impact.

### Data analysis

The data analysis phase required time to prepare data, including the physiological data export files from Whoop technology, and the survey data recorded and collected via Qualtrics. Exploratory stratification by sex was conducted to examine potential differences in symptom response and physiological changes between male and female participants. Prior research has documented sex-specific patterns in PTSD prevalence, symptom expression, and recovery trajectories (Olff, 2017), as well as differences in autonomic regulation and stress biomarkers such as HRV (Nater and Rohleder, 2009). While the sample size was not powered for inferential sex comparisons, these subgroup analyses offer initial insights and inform future hypothesis generation for larger studies. Descriptive statistics were computed to establish sample and preprogram characteristics and to determine whether the data met the statistical assumptions required for paired samples *t* tests, including tests for independence, the absence of significant outliers, homogeneity of variances, and the normality of the dependent variables. All analyses were conducted in SPSS version 28 (IBM Corporation, 2024). Paired samples *t* tests were then used using the GAD-7, PCL-5, and PHQ-8 scores as the dependent variable and time (i.e., Time Period 1, Time Period 2, and Time Period 3) as the within-subject variable. These tests were conducted at the *p* ≤ 0.05 level of statistical significance. The results of this analysis were used as the basis to determine whether participation in Operation Surf’s program resulted in statistically significant changes in the PTSD symptoms, depression, or anxiety of veterans. In addition to statistical significance, clinical analysis was also employed specific to each survey using diagnostic scoring methods to understand if any clinically significant changes occurred.

This study employed paired sample *t*-tests to evaluate changes over time in both psychological symptom scores and physiological biomarkers. This analytic approach was appropriate given the study’s design and objectives: each outcome was assessed within-subject at specific timepoints (pre-, post-, and 30-day follow-up), and analyses were only conducted when data were available for both timepoints. Regression analyses were not used, as the research questions were not designed to test predictive relationships but rather to explore change within domains. Psychological and physiological outcomes were treated as separate, parallel lines of inquiry, consistent with the goals of a feasibility study.

For the physiological data, paired sample *t* tests were then conducted using the physiological biomarker data as the dependent variable and time (i.e., Time Period 1, Time Period 2, and Time Period 3) as the within-subject variable. These tests were conducted at the *p* ≤ 0.05 level of statistical significance. The result of this analysis was used as the basis to determine whether participation in Operation Surf resulted in statistically significant changes in physiological biomarkers of veterans. Additional analysis was conducted using the mean of the point in times for individuals, and the minimums, maximums, and means for all individual participants. Analysis was also conducted by cohort and by gender to segment the data in an effort to further understanding. The paired sample *t*-tests for differences between paired measurements for this research met all assumptions necessary (i.e., subjects were independent, measurements for one subject did not affect measurement for any other subject, each of the paired measurements were obtained from the same subject, and measured differences were normally distributed).

### Sample size justification

The sample size for this feasibility study was determined based on practical, methodological, and resource considerations. Given the exploratory nature and novelty of incorporating wearable technology for physiological measurement in surf therapy, no previous research provided effect sizes suitable for *a priori* power analysis. Therefore, the sample size was established primarily through convenience sampling, including all eligible military veterans enrolled in the Operation Surf therapy program within a one-year timeframe (May 2021 to May 2022). Operation Surf planned to hold six weeklong events during the May 2021 through May 2022 timeframe, each hosting eight military veteran participants, for a sample size of *n* = 48 participants. One of the events changed plans and invited seven active duty injured military participants, and one military veteran, therefore the sample size was reduced to *n* = 41 (Figure 1). A period of 1 year was considered a reasonable duration to collect data on participants within resource constraints typical of feasibility studies. This approach was deemed appropriate given logistical constraints, participant availability, and the goal of maximizing ecological validity in this unique real-world intervention setting, while also monitoring financial cost of conducting the programs and collecting the data. This study was also the first to integrate physiological biomarker data with psychological assessments, building upon previous work which relied solely on self-report psychological assessments and Operation Surf (Crawford, 2019), providing a foundational basis for future research in this area.

### Eligibility and selection

All study participants were selected through Operation Surf’s standard application process for their weeklong therapeutic surfing events. To be eligible, applicants were required to: (1) be active duty, active reserve, or honorably discharged veterans of the U.S. Armed Forces; (2) have a service-connected injury, either visible or invisible (e.g., PTSD); (3) have served in at least one combat deployment; and (4) have served post-9/11. Applicants also needed to complete the full online application, including short essay questions, and commit to attending the full weeklong program. Operation Surf staff reviewed submissions based on service eligibility, readiness to participate, and program availability. Individuals with acute psychiatric instability (e.g., current suicidal ideation requiring inpatient care), active substance dependence, or physical conditions that posed a safety risk in open water were excluded.

All veterans who were selected and attended Operation Surf events during the study period (May 2021–May 2022) were invited to participate in the research study, using a nonprobability sampling strategy based on availability and interest.

PTSD diagnosis for inclusion in the program was self-reported by applicants as part of the Operation Surf application process and was not independently verified by the research team through medical records or clinician documentation. However, many participants referenced prior diagnoses or treatment history in their application narratives, and the program primarily targeted veterans with known PTSD symptoms.

PTSD was selected as the primary inclusion criterion because Operation Surf specifically targets veterans with service-connected trauma and post-traumatic stress symptoms. While anxiety and depression are frequently comorbid with PTSD, they were not used as standalone inclusion criteria in order to preserve alignment with the program’s mission and population. This approach allowed for the evaluation of symptom changes across related domains without excluding veterans who may not have met criteria for a secondary diagnosis.

## Results

A total of 41 military veterans with PTSD were enrolled in the study (*n* = 41). In addition to a PTSD diagnosis, military veteran participants reported moderate levels of anxiety, as indicated by Generalized Anxiety Disorders 7-item measure (GAD-7) mean score of 10.29 (*n* = 32) at Time Point 1, prior to the event. For further details on descriptive statistics, see Tables 2–4.

**Table 2:** Descriptive statistics for depression, PTSD symptoms, anxiety, deep sleep, REM sleep, and HRV, at three points in time (PHQ-8, PCL-5, GAD-7).

Survey	Gender	Pair		*M*	*n*	*SD*
PHQ-8	All participants	1	Time Point 1	11.12	17	4.68
			Time Point 2	6.18	17	4.13
PHQ-8	All participants	2	Time Point 2	8.44	18	5.48
			Time Point 3	9.11	18	6.06
PHQ-8	All participants	3	Time Point 1	12.15	20	5.32
			Time Point 3	8.50	20	4.51
PHQ-8	Female	1	Time Point 1	12.50	4	2.65
			Time Point 2	6.25	4	2.63
PHQ-8	Female	2	Time Point 2	10.83	6	7.08
			Time Point 3	10.50	6	7.26
PHQ-8	Female	3	Time Point 1	15.60	5	4.16
			Time Point 3	10.60	5	2.41
PHQ-8	Male	1	Time Point 1	10.69	13	5.15
			Time Point 2	6.15	13	4.58
PHQ-8	Male	2	Time Point 2	7.25	12	4.35
			Time Point 3	8.42	12	5.58
PHQ-8	Male	3	Time Point 1	11.00	15	5.28
			Time Point 3	7.80	15	4.89
PCL-5	All participants	1	Time Point 1	44.82	22	13.77
			Time Point 2	27.86	22	13.81
PCL-5	All participants	2	Time Point 2	28.47	19	14.70
			Time Point 3	27.11	19	16.00
PCL-5	All participants	3	Time Point 1	43.35	20	12.55
			Time Point 3	28.30	20	15.65
PCL-5	Female	1	Time Point 1	51.14	7	13.51
			Time Point 2	26.71	7	11.13
PCL-5	Female	2	Time Point 2	34.43	7	14.25
			Time Point 3	32.14	7	15.64
PCL-5	Female	3	Time Point 1	51.33	6	6.77
			Time Point 3	37.17	6	12.64
PCL-5	Male	1	Time Point 1	41.87	15	13.30
			Time Point 2	28.40	15	15.24
PCL-5	Male	2	Time Point 2	25.00	12	14.39
			Time Point 3	24.17	12	16.12
PCL-5	Male	3	Time Point 1	39.93	14	13.06
			Time Point 3	24.50	14	15.64
GAD-7	All participants	1	Time Point 1	12.44	16	5.49
			Time Point 2	5.06	16	3.94
GAD-7	All participants	2	Time Point 2	6.46	13	5.50
			Time Point 3	7.77	13	5.96
GAD-7	All participants	3	Time Point 1	11.59	17	5.09
			Time Point 3	8.06	17	4.85
GAD-7	Female	1	Time Point 1	13.00	3	4.36
			Time Point 2	6.00	3	4.00
GAD-7	Female	2	Time Point 2	9.75	4	8.18
			Time Point 3	12.00	4	6.22
GAD-7	Female	3	Time Point 1	13.00	5	3.39
			Time Point 3	8.80	5	2.86
GAD-7	Male	1	Time Point 1	12.31	13	5.86
			Time Point 2	4.85	13	4.06
GAD-7	Male	2	Time Point 2	5.00	9	3.54
			Time Point 3	5.89	9	5.09
GAD-7	Male	3	Time Point 1	11.00	12	5.67
			Time Point 3	7.75	12	5.56
Deep Sleep	All participants	1	Time Point 1	1.27	26	0.44
			Time Point 2	1.39	26	0.40
Deep Sleep	All participants	2	Time Point 2	1.37	29	0.39
			Time Point 3	1.28	29	0.33
Deep Sleep	All participants	3	Time Point 1	1.27	26	0.44
			Time Point 3	1.29	26	0.34
Deep Sleep	Female	1	Time Point 1	1.16	9	0.56
			Time Point 2	1.47	9	0.41
Deep Sleep	Female	2	Time Point 2	1.43	10	0.41
			Time Point 3	1.22	10	0.41
Deep Sleep	Female	3	Time Point 1	1.16	9	0.56
			Time Point 3	1.24	9	0.43
Deep Sleep	Male	1	Time Point 1	1.32	17	0.37
			Time Point 2	1.35	17	0.40
Deep Sleep	Male	2	Time Point 2	1.35	19	0.38
			Time Point 3	1.31	19	0.28
Deep Sleep	Male	3	Time Point 1	1.32	17	0.37
			Time Point 3	1.32	17	0.30
REM	All participants	1	Time Point 1	1.48	25	0.44
			Time Point 2	1.59	25	0.51
REM	All participants	2	Time Point 2	1.56	29	0.53
			Time Point 3	1.37	29	0.47
REM	All participants	3	Time Point 1	1.48	25	0.44
			Time Point 3	1.46	25	0.41
REM	Female	1	Time Point 1	1.49	8	0.35
			Time Point 2	1.75	8	0.46
REM	Female	2	Time Point 2	1.70	10	0.55
			Time Point 3	1.40	10	0.44
REM	Female	3	Time Point 1	1.49	8	0.35
			Time Point 3	1.50	8	0.42
REM	Male	1	Time Point 1	1.47	17	0.49
			Time Point 2	1.52	17	0.54
REM	Male	2	Time Point 2	1.49	19	0.51
			Time Point 3	1.35	19	0.49
REM	Male	3	Time Point 1	1.47	17	0.49
			Time Point 3	1.44	17	0.42
HRV	All participants	1	Time Point 1	41.94	26	27.90
			Time Point 2	37.05	26	23.22
HRV	All participants	2	Time Point 2	36.88	28	22.66
			Time Point 3	39.31	28	20.84
HRV	All participants	3	Time Point 1	41.94	26	27.90
			Time Point 3	40.39	26	21.25
HRV	Female	1	Time Point 1	31.02	9	13.68
			Time Point 2	27.25	9	12.35
HRV	Female	2	Time Point 2	29.38	10	13.44
			Time Point 3	30.43	10	10.06
HRV	Female	3	Time Point 1	31.02	9	13.68
			Time Point 3	30.70	9	10.63
HRV	Male	1	Time Point 1	47.72	17	31.95
			Time Point 2	42.24	17	26.15
HRV	Male	2	Time Point 2	41.06	18	25.86
			Time Point 3	44.24	18	23.75
HRV	Male	3	Time Point 1	47.72	17	31.95
			Time Point 3	45.51	17	23.84

**Table 3:** Paired sample statistics for depression, PTSD symptoms, anxiety, REM sleep, deep sleep, and HRV at three points in time.

Survey	Segmentation	Pair		*SMD*	*SD*	*t*	*df*	Two-sided *p*
PHQ-8	All participants	1	Time Point 1—Time Point 2	4.94	4.66	4.38	16	< 0.01
		2	Time Point 2—Time Point 3	˗0.67	5.77	˗0.49	17	0.63
		3	Time Point 1—Time Point 3	3.65	5.32	3.07	19	< 0.01
PHQ-8	Female	1	Time Point 1—Time Point 2	6.25	0.96	13.06	3	< 0.01
		2	Time Point 2—Time Point 3	0.33	5.09	0.16	5	0.88
		3	Time Point 1—Time Point 3	5.00	3.94	2.84	4	0.05
PHQ-8	Male	1	Time Point 1—Time Point 2	4.54	5.29	3.10	12	< 0.01
		2	Time Point 2—Time Point 3	˗1.17	6.24	˗0.65	11	0.53
		3	Time Point 1—Time Point 3	3.20	5.76	2.15	14	0.05
PCL-5	All participants	1	Time Point 1—Time Point 2	16.95	14.17	5.61	21	< 0.01
		2	Time Point 2—Time Point 3	1.37	16.84	0.35	18	0.73
		3	Time Point 1—Time Point 3	15.05	14.53	4.63	19	< 0.01
PCL-5	Female	1	Time Point 1—Time Point 2	24.43	13.55	4.77	6	< 0.01
		2	Time Point 2—Time Point 3	2.29	22.43	0.27	6	0.80
		3	Time Point 1—Time Point 3	14.17	15.48	2.24	5	0.08
PCL-5	Male	1	Time Point 1—Time Point 2	13.47	13.48	3.87	14	< 0.01
		2	Time Point 2—Time Point 3	0.83	13.74	0.21	11	0.84
		3	Time Point 1—Time Point 3	15.43	14.70	3.93	13	< 0.01
GAD-7	All participants	1	Time Point 1—Time Point 2	7.38	5.00	5.89	15	< 0.01
		2	Time Point 2—Time Point 3	˗1.31	5.02	˗0.94	12	0.37
		3	Time Point 1—Time Point 3	3.53	6.49	2.24	16	0.04
GAD-7	Female	1	Time Point 1—Time Point 2	7.00	1.73	7.00	2	0.02
		2	Time Point 2—Time Point 3	˗2.25	4.57	˗0.98	3	0.40
		3	Time Point 1—Time Point 3	4.20	4.44	2.12	4	0.10
GAD-7	Male	1	Time Point 1—Time Point 2	7.46	5.55	4.85	12	< 0.01
		2	Time Point 2—Time Point 3	˗0.89	5.42	˗0.49	8	0.64
		3	Time Point 1—Time Point 3	3.25	7.34	1.53	11	0.15
Deep sleep	All participants	1	Time Point 1—Time Point 2	−0.12	0.36	−1.74	25	0.10
		2	Time Point 2—Time Point 3	0.09	0.23	2.14	28	0.04
		3	Time Point 1—Time Point 3	−0.03	0.26	−0.50	25	0.62
Deep sleep	Female	1	Time Point 1—Time Point 2	−0.31	0.32	−2.89	8	0.02
		2	Time Point 2—Time Point 3	0.21	0.24	2.70	9	0.03
		3	Time Point 1—Time Point 3	−0.08	0.33	−0.71	8	0.50
Deep sleep	Male	1	Time Point 1—Time Point 2	−0.03	0.35	−0.30	16	0.77
		2	Time Point 2—Time Point 3	0.03	0.21	0.67	18	0.51
		3	Time Point 1—Time Point 3	0.00	0.23	0.03	16	0.97
REM	All participants	1	Time Point 1—Time Point 2	−0.12	0.38	−1.50	24	0.15
		2	Time Point 2—Time Point 3	0.20	0.54	1.96	28	0.06
		3	Time Point 1—Time Point 3	0.02	0.42	0.21	24	0.83
REM	Female	1	Time Point 1—Time Point 2	−0.26	0.36	−2.03	7	0.08
		2	Time Point 2—Time Point 3	0.30	0.42	2.22	9	0.05
		3	Time Point 1—Time Point 3	−0.01	0.42	−0.06	7	0.96
REM	Male	1	Time Point 1—Time Point 2	−0.05	0.39	−0.51	16	0.62
		2	Time Point 2—Time Point 3	0.14	0.60	1.05	18	0.31
		3	Time Point 1—Time Point 3	0.03	0.42	0.29	16	0.77
HRV	All participants	1	Time Point 1—Time Point 2	−4.89	10.48	2.38	25	0.03
		2	Time Point 2—Time Point 3	−2.42	7.71	−1.66	27	0.11
		3	Time Point 1—Time Point 3	−1.56	12.00	−0.66	25	0.51
HRV	Female	1	Time Point 1—Time Point 2	3.77	5.88	1.92	8	0.09
		2	Time Point 2—Time Point 3	1.06	9.23	0.36	9	0.73
		3	Time Point 1—Time Point 3	−0.32	7.34	−1.31	8	0.90
HRV	Male	1	Time Point 1—Time Point 2	5.49	12.38	1.83	16	0.09
		2	Time Point 2—Time Point 3	3.18	6.90	1.96	17	0.07
		3	Time Point 1—Time Point 3	−2.21	14.03	−0.65	16	0.53

**Table 4:** Descriptive statistics raw data scores psychological surveys at three points in time (prior to paired sample calculation).

Psychological survey	Time Point 1 (*n*)	Time Point 1 Score	Time Point 2 (*n*)	Time Point 2 Score	Time Point 3 (*n*)	Time Point 3 Score
GAD7	30	10.29	21	4.69	21	7.47
PCL5	32	38.58	26	25.23	23	21.78
PHQ8	32	11.19	22	5.64	26	6.89

### Psychological outcomes

#### Anxiety

Across participants, GAD-7 scores changed significantly from prior to the event compared to immediately after the event and at 30 days following the event. Specifically, there was a 59% reduction in general anxiety immediately after the event, using the before the event (mean = 12.44) to immediately after the event (mean = 5.06) with a mean difference of 7.38 (*p* ≤ 0.01). From before the event to 30 days after the event, anxiety reduced by 30%, with mean scores decreasing from 11.59 to 8.06, a mean difference of 3.53 (*p* ≤ 0.05). These means reflect only those participants who completed the GAD-7 at both time points for each comparison; while more participants completed the survey at individual time points, paired-sample analyses include only those with matched responses to assess within-subject change. For further details see Table 3.

##### Gender analysis

Female participants experienced a 54% reduction in general anxiety, with GAD-7 scores dropping from 13.00 before the event to 6.00 immediately after the event, a difference of 7.00 (*p* ≤ 0.05; see Tables 2, 3). Changes between Time Points 2 and 3, and 1 and 3, were not statistically significant for females. Male participants saw a 61% decrease in general anxiety, with GAD-7 scores falling from 12.31 before the event to 4.85 immediately after the event, a difference of 7.46 (*p* ≤ 0.01; see Tables 2, 3). Changes between Time Points 2 and 3, and 1 and 3, were not significant for males.

### Clinical results analysis

#### Anxiety

All 41 military veterans agreed to participate in the GAD-7 survey, with completion rates of 73% (*n* = 30) before the event and 51% (*n* = 21) immediately after the event and 51% (*n* = 21) 30 days after the event. Prior to the event, 40% of participants (12/30) scored in the highest classification of severe anxiety (i.e., scoring between 15–21 on a 21-point maximum). Immediately following the event, 10% of participants (2/21) scored in the highest classification of severe anxiety, indicating a 30% reduction in severe anxiety severity scores immediately following the event. At 30 days following the event, 14% of participants (3/21) scored in the highest classification of severe anxiety, indicating a 26% reduction in severe anxiety scores at 30 days following the event when compared to prior to the event (see Table 5).

**Table 5:** Anxiety Severity scoring (GAD-7) at three points in time.

Total severity score	Interpretation of total score	T1 (*n* = 30)	T2 (*n* = 21)	T3 (*n* = 21)
0–4	Minimal anxiety	4	8	4
5–9	Mild anxiety	5	8	10
10–14	Moderate anxiety	9	3 (14.29%) 15.71% reduction	4 (19.05%) 10.95% reduction
15–21	Severe anxiety	12	2 (9.52%) 30.48% reduction	3 (14.29%) 25.71% reduction

Prior to the event, 30% of participants (9/30) scored in the next highest classification of moderate anxiety (i.e., scoring between 10–14 on a 21-point maximum). Immediately after the event, 14% (3/21) scored as having moderate anxiety, indicating a 16% reduction in moderate anxiety scores immediately following the ocean therapy event. At 30 days following the event, 19% (4/21) scored as having moderate anxiety, indicating an 11% reduction in moderate anxiety 30 days after the event when compared to before the event (see Table 5).

#### Depression

All 41 military veterans agreed to participate in the PHQ-8 survey, with completion rates of 78% (*n* = 32) before the event (Time Point 1), 54% (*n* = 22) immediately following the event (Time Point 2), and 63% (*n* = 26) at 30 days following the event (Time Point 3; Table 2). For analyses requiring paired data (e.g., paired samples t-tests on PHQ-8 scores), only participants who completed surveys at both relevant time points were included: *n* = 17 for Time Points 1 and 2, *n* = 18 for Time Points 2 and 3, and *n* = 20 for Time Points 1 and 3. Across participants, scores showed statistically significant reductions in depression following participation in the ocean surf therapy event. Specifically, participants scored a 44% reduction in depression prior to the event (mean = 11.12) to immediately after the event (mean = 6.18) with a paired difference mean reduction of 4.94 (*p* ≤ 0.01, *n* = 17). 30 days following the event depression reduced 30%, with mean scores decreasing from 12.15 to 8.50, a mean difference of 3.65 (*p* ≤ 0.01, *n* = 20). The change between the second and third time points was not statistically significant. For further details, see Table 2.

##### Gender analysis

Female participants experienced a 50% reduction in depression, with PHQ-8 paired sample t-test scores dropping from 12.50 before the event to 6.25 immediately following the event, a difference of 6.25 (*p* ≤ 0.01, *n* = 4; see Tables 2, 3). Females scored a 32% reduction in depression 30 days following the event, with PHQ-8 mean scores dropping from 15.60 at Time Point 1 to 10.60 at Time Point 3, resulting in a mean difference of 5.0 (*p* ≤ 0.05, *n* = 5). The changes from the Time Point 2 to the Time Point 3 were not statistically significant for females.

Male participants scored a 42% reduction in depression immediately following the event, with PHQ-8 scores decreasing from 10.69 at Time Point 1 to 6.15 at Time Point 2, a paired mean difference of 4.54 (*p* ≤ 0.01, *n* = 13; see Tables 2, 3). At 30 days post-event, a reduction of 29% was observed, with scores lowering from 11.00 at Time Point 1 to 7.80 at Time Point 3, a paired mean difference of 3.20 (*p* ≤ 0.05, *n* = 15). The paired *t*-tests between Time Point 2 and Time Point 3 was not statistically significant.

### Clinical results analysis

Using the PHQ-8 to measure depression, as indicated by the PHQ-8 depression scale scoring guidelines, 6% of participants (2/32) scored the highest classification of severe depression (scoring between 20–24 on a 24-point maximum) prior to the event, with the number reporting severe depression reducing to 5% (1/22) immediately after the event. After 30 days following the event there was 4% of participants (1/26) scoring severe depression (see Table 6).

**Table 6:** Depression (PHQ-8) severity scoring at three points in time.

Total severity score	Interpretation of total score	T1 (*n* = 32)	T2 (*n* = 22)	T3 (*n* = 26)
0–4	None	3	9	8
5–9	Mild depression	7	6	7
10–14	Moderate depression	6	6	8
15–19	Moderately severe depression	14	0	2
20–24	Severe depression	2	1	1

44% of participants (14/32) had the second highest classification of moderately severe depression (i.e., 15–19 total score on a 24-point maximum) before the event, while zero participants scored moderately severe depression immediately following the event, showcasing a reduction in 44% after participating in an ocean surf therapy event (see Table 6). 30 days after the event, 8% of participants (2/26) had the second highest classification of moderately severe depression, indicating a 36% reduction in depression when compared to prior to the ocean surf therapy event. Combining the two highest classifications of depression, severe and moderately severe depression, there was a total of 16 participants (16/32) falling into these categories, while this number reduced to 1 participant (1/22) immediately after the event, while only 3 participants (3/26) fell into these higher classifications of depression when surveyed 30 days after the ocean surf therapy event.

#### PTSD

All 41 military veterans agreed to participate in the PCL-5 survey, with completion rates of 78% (32/41=0.78) before the event (Time Point 1), 63% (*n* = 26) immediately after the event (Time Point 2) and 56% (*n* = 23) 30 days after the event (Time Point 3). Across participants, scores showed statistically significant reductions in PTSD following participation in the ocean surf therapy event (see Table 3). Using paired sample *t*-tests, there was a 38% reduction in PTSD symptoms from before the event (mean = 44.82) to immediately after the event (mean = 27.86), with a mean difference of 16.95 (*p* ≤ 0.01, *n* = 22). Paired sample t test showed a 35% reduction from before the event (mean = 43.35) compared to 30 days following the event (mean = 28.30), with a mean difference of 15.05 (*p* ≤ 0.01, *n* = 20).

##### Gender analysis

Female participants survey scores reported a 48% reduction in PTSD symptoms immediately following the event, with a PCL-5 paired sample t-test mean score of 51.14 prior to the event, and a 26.71 mean score immediately following the event (Time Point 2), a paired mean difference of 24.43 (*p* ≤ 0.01, *n* = 7). Males reported a 32% reduction in PTSD symptoms immediately following the event, reporting a 41.87 mean score prior to the event, and a 28.40 mean score immediately following the event, a paired mean difference of 13.47 (*p* ≤ 0.01, *n* = 15). At 30 days following the event, males reported a 39% reduction in PTSD symptoms, with a mean score of 39.93 prior to the event and a 24.50 mean score at 30 days following event, resulting in a paired mean difference of 15.43 (*p* ≤ 0.01, *n* = 14). This score, Men’s PTSD symptoms, is unique from previous research in that the statistically significant reduction continued to increase in the 30 days following the event. The addition of GroupMe text chats for all participants and weekly zoom meetings at the conclusion of the weeklong event allowing for communication and connection will be discussed later in the discussion section.

### Clinical analysis

Using the PCL-5 as a measure of PTSD, 41 military veterans were asked to participate in the PCL-5, and all 41 agreed. Of the 41 participants, 78% (*n* = 32) completed the survey prior to the event (Time Point 1); 63% (*n* = 26) completed the survey immediately after the event (Time Point 2), and 56% (*n* = 23) completed the survey at 30 days after the event (Time Point 3; See Table 3).

**Figure 2:**
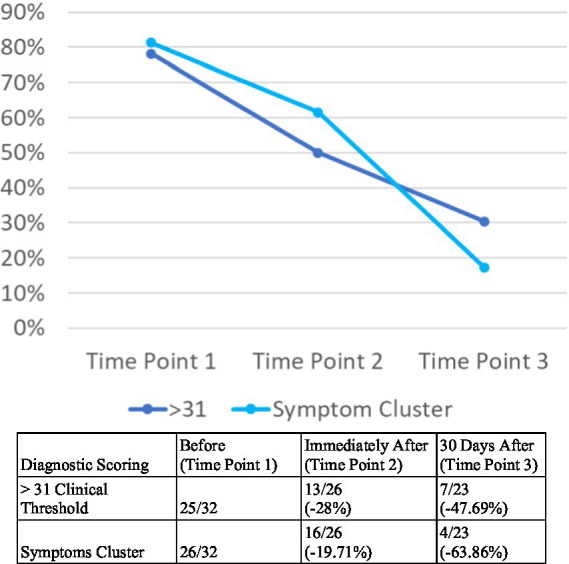
PTSD symptoms diagnostic scoring clinical analysis at three points in time (PCL-5).

For this study, a cutoff score of 31 was selected, deemed reasonable based on current psychometric research (National Center for PTSD, 2017). Evidence for the PCL for DSM-IV suggests 5 points as a minimum threshold for determining whether an individual has responded to treatment and 10 points as a minimum threshold for determining whether the improvement is clinically meaningful. Preliminary guidance for the PTSD Checklist for DSM-5 (PCL-5) shows thresholds are expected to be similar for the PCL-5, but specific change scores are still being determined. Until new information is available, it is recommended to follow the DSM-IV guidelines (National Center for PTSD, 2017). Prior to the event, 78% (25/32) participants had above a clinical threshold of PTSD symptoms by severity measure (see Table 7 and Figure 2). Immediately after the event, 50% (13/26) participants had above a clinical threshold of PTSD symptoms, which was a reduction of 28%. Participants reported a 48% reduction in PTSD symptoms at 30 days following the event, with 30% (7/23) scoring above clinically meaningful threshold, a reduction of 10 points (see Figure 2).

**Table 7:** PTSD (PCL-5) clinical scoring table.

Pair		*n*	10 + Point clinically significant change	5–10 Point meaningful change	< 5 Point change
1	Time Point 1—Time Point 2	22	17	1	4
2	Time Point 1—Time Point 3	21	15	2	4

Using the symptoms cluster approach, prior to the event, 81% (26/32) participants scored above a clinical threshold level for PTSD symptoms. Immediately after the event, 62% (16/26) reported an above clinical threshold for PTSD, a reduction of 20% compared to before the event. After the 30 days following the event, 17% of participants (4/23) reported an above clinical threshold score, a 64% reduction in participants with probable PTSD compared to prior to the event (see Figure 2).

On an individual basis, 86% of participants (19/22) who completed the survey before the event and immediately after the event, saw a reduction in their severity score (Ossie, 2023). One individual (4.55%, 1/22) had a 5–10-point reliable reduction in total score, while an additional 77% participants (17/22) had a 10–20 point clinically meaningful reduction in score immediately following the event (see Table 6).

For those participants who completed the survey before the event and 30 days following the event, 90% (19/21) showed a reduction in their total overall score (Ossie, 2023). Two individuals (10%, 2/21) had a 5–10-point meaningful reduction in total score and an additional 71% participants (15/21) reported a 10–20 clinically meaningful reduction in total score (see Table 7).

#### Physiological biomarkers

A total of 33 veterans were contacted for participation in this data collection effort, and all 33 agreed to participate. The slightly lower number of Whoop band wearers (*n* = 33) compared to the survey participants (*n* = 41) was due to a supply chain issue with Whoop straps; Whoop was unable to fulfill the November 2021 order for hardware. Fortunately, the supply chain issue was resolved for 2022. Numerous physiological biomarkers were collected via Whoop band wearable technology; for this research study, sleep data, specifically deep sleep and REM sleep, along with HRV were chosen for analysis.

#### Deep sleep

Of the 33 contacted for physiological biomarker participation, 82% of participants (*n* = 27) logged data scores prior to the event. Using paired sample t-tests for all participants comparing before, during, and after the event, only the pair 2 comparison of during and after the event yielded a statistically significant computation, with deep sleep falling 7% in the 30 days following the event (*p* ≤ 0.05). During the event (Time Point 2) all participants scored a mean 1.37 h of deep sleep, with the 30 days following the event mean dropping to 1.28, a paired mean difference of 0.09 (*n* = 29).

##### Gender analysis

Females increased deep sleep 27% during the event compared to before the event, reporting a mean deep sleep score of 1.47 h, compared to 1.16 h prior, a paired mean difference of 0.31 (*p* ≤ 0.05; *n* = 9). Additionally, females deep sleep fell 15% reporting for the 30 days following the event compared to during the event, reporting a mean score of 1.43 h deep sleep during the event and 1.22 h of deep sleep mean for the 30 days following, a paired mean difference of 0.21 (*p* ≤ 0.05; *n* = 10).

#### REM sleep

REM sleep for females fell 18% following the event compared to during the event, dropping from 1.70 h mean score during, to 1.40 h mean score for the 30 days following the event (*p* < 0.05; *n* = 10), however, paired sample *t*-tests did not yield any statistically significant changes for all participants.

#### HRV

Across all participants, HRV mean scores changed significantly from before to during the program (MD = –4.89, *p* < 0.05). Specifically, HRV decreased 41.94 to 37.05, a decrease of 12%. HRV paired sample t test scores did not change with statistical significance for other time comparisons. For a complete list of longitudinal program results, see Tables 2, 3.

## Discussion

Surf therapy incorporates a multifaceted approach that integrates social, psychological, physiological, and environmental components through physical activity in outdoor settings. Prior research suggests that natural environments may offer unique mental health benefits beyond physical activity alone, particularly in reducing tension, anger, and depression (Thompson Coon et al., 2011; Walter et al., 2019). Additionally, neuroscience research has highlighted therapeutic responses associated with proximity to water (Nichols, 2014).

Emerging evidence indicates that surf therapy may positively affect psychological and psychosocial outcomes among veterans, including symptoms of PTSD (Benninger et al., 2020; Crawford, 2016; Walter et al., 2019). However, empirical research on surf therapy remains limited in scope and methodological rigor. This study contributes to the field by leveraging wearable technology to collect objective physiological data in conjunction with validated psychological instruments, providing a more holistic understanding of intervention effects.

Findings demonstrated statistically and clinically significant reductions in anxiety, depression, and PTSD symptoms immediately after the intervention and at the 30-day follow-up. These outcomes support the potential of surf therapy to improve short-term psychological functioning among military veterans. Physiological metrics—particularly sleep and HRV—provided objective corroboration of psychological change, and revealed notable sex-specific effects, especially in sleep quality for female participants.

The supportive group environment and structured daily activities, including the novel use of GroupMe text chats and weekly Zoom check-ins, may have reinforced social connectedness and mitigated isolation. For male participants, PTSD symptom reductions continued to improve 30 days post-intervention, suggesting potential lasting benefits tied to camaraderie and peer connection. These findings align with prior research suggesting that surf therapy may serve as a meaningful social intervention that supports reintegration and well-being (Benninger et al., 2020; Rogers et al., 2014).

Improvements in anxiety and depression were most prominent immediately after the event, with partial attenuation by 30 days. This trajectory supports existing literature on nature-based interventions and suggests the possibility of “dose effects” in surf therapy (Walter et al., 2019; Walter et al., 2023). Future research should examine optimal dosing schedules and compare the effects of single-week interventions with longer or more frequent programs. For example, Operation Surf’s three-month program in San Luis Obispo, California offers a promising model for longitudinal investigation.

Although the study was not powered for between-group comparisons, sex-stratified analyses revealed some noteworthy trends. Female participants tended to show larger immediate reductions in depression and PTSD symptoms, while male participants demonstrated continued improvement at 30 days, particularly for PTSD. This delayed response in men may reflect the impact of post-program peer support mechanisms (e.g., GroupMe chats and Zoom meetings), which may have prolonged social engagement. In terms of physiological data, females showed a more pronounced increase in deep and REM sleep during the intervention period. These findings are consistent with prior research indicating sex differences in emotional regulation, stress recovery, and sleep architecture following trauma exposure (Olff, 2017). While exploratory, these trends highlight the importance of considering sex as a potential moderator in future studies of surf and nature-based therapies.

Participants frequently described immersive experiences akin to flow state, a psychological construct characterized by deep focus, enjoyment, and presence (Csikszentmihalyi, 1990). This sense of “therapeutic immersion” may interrupt cycles of rumination and hyperarousal in PTSD. Given the parallels between flow theory and behavioral activation, future studies should incorporate validated flow state measures such as the Flow State Scale-2 (FSS-2) or Dispositional Flow Scale (DFS-2) to quantify this mechanism (Jackson and Marsh, 1996; Jackson and Eklund, 2002).

Sleep findings were promising but preliminary, particularly among female participants who showed increased deep and REM sleep during the intervention. Conversely, HRV decreased during the intervention, which may reflect the physiological demands of surfing rather than stress alleviation. These biomarkers provide new insight into the physiological dynamics of therapy and underscore the value of wearable technology in mental health research.

The wearable biometrics in this study were not used as standalone clinical outcomes but as adjunctive measures to provide additional context to self-reported changes in mental health symptoms. The rationale for including HRV and sleep architecture (REM and deep sleep) was based on their established association with PTSD symptomatology, autonomic regulation, and emotional processing. Their inclusion enhances the interpretability of outcomes and supports future research directions that integrate subjective and objective data. Patient-reported outcomes (GAD-7, PHQ-8, PCL-5) remained the primary basis for evaluating program effectiveness.

Although statistically significant changes in HRV and sleep were observed, these physiological markers were not directly linked to self-reported clinical outcomes in this study. We did not assess correlations between wearable-derived metrics and changes in anxiety, depression, or PTSD scores, and therefore cannot conclude whether the biometric changes were clinically meaningful. These data are best interpreted as exploratory signals that support the feasibility of physiological monitoring and may inform future research on potential mechanisms of change in nature-based therapies.

Limitations of this study include its use of nonprobability sampling, modest sample size, lack of a control group, and reliance on self-reported data. The inability to isolate treatment effects from other concurrent therapies limits causal inferences. While wearable technology offered a novel method of data collection, it too warrants further validation in clinical research settings. This study should be considered a feasibility trial that sets the stage for future randomized controlled investigations.

Another limitation is that HRV was not adjusted for age or sex in this analysis. Although HRV is known to vary by these demographic factors, the study used a within-subjects design (paired-sample *t*-tests) to evaluate changes over time, rather than comparing individuals against one another. Because the primary objective was to assess within-person change in a feasibility context, age and sex were not included as covariates. However, these variables may influence HRV interpretation, particularly at baseline, and future studies should incorporate age- and sex-adjusted models to improve precision and generalizability.

Additionally, the timing of symptom assessments presents a limitation. While GAD-7 and PHQ-8 were administered at intervals that allowed for non-overlapping two-week recall windows, the PCL-5 has a one-month recall period. As a result, the PCL-5 administered immediately post-intervention (Time Point 2) may include symptoms that occurred during the pre-intervention period, making it more difficult to isolate the immediate effects of the intervention on PTSD symptoms. This limitation does not apply to the Time Point 3 PCL-5 assessment, which reflects a distinct post-intervention month.

Another potential limitation is the risk of a Hawthorne effect, in which participants may have altered their behavior or responses simply because they knew they were part of a research study. This could have led to inflated improvements in self-reported outcomes, particularly for measures like depression, anxiety, or PTSD symptoms. Although this effect is difficult to quantify, it is an inherent limitation in open-label intervention studies, especially those involving intensive, community-based experiences. The use of physiological data (e.g., HRV, sleep) provided a partial counterbalance by offering objective, non-self-reported metrics, but even these may have been indirectly influenced by increased participant engagement or expectancy effects. Future studies could consider the inclusion of control or comparison groups to help disentangle true intervention effects from participant reactivity. The author’s involvement in the Operation Surf program may have introduced potential bias into participant responses, particularly for self-reported outcomes. Although the research team was not involved in clinical instruction or therapeutic delivery, their presence during the program could have led to social desirability bias. To reduce this risk, all surveys were anonymized and self-administered via the Qualtrics platform, and participants were repeatedly reminded—both in writing and verbally—that their participation was voluntary, responses were confidential and would have no bearing on their access to services or relationships with program staff. These measures were designed to encourage honest reporting and mitigate undue influence. Nonetheless, future studies may benefit from additional protections such as blinding data analysts or using independent researchers for follow-up assessment.

Another limitation is the risk of survivorship bias, which may have influenced the results due to incomplete follow-up survey data. Participants who completed post-intervention and 30-day surveys may represent a more engaged or positively responsive subset, while those with less improvement or lower satisfaction may have been less likely to complete follow-up assessments. This could lead to an overestimation of treatment effects, particularly for self-reported symptom change. Although follow-up rates were within the expected range for community-based feasibility studies, this potential bias underscores the importance of maximizing retention in future trials and examining the characteristics of non-responders.

An additional limitation of this study is the lack of detailed socio-demographic and clinical data. While sex, age, and deployment history were recorded, variables such as education level, occupation status, disability rating, substance use, and current PTSD treatment or medication use were not collected. This limits our ability to interpret differential treatment responses or generalize findings across veteran subgroups. Future studies should incorporate comprehensive baseline demographic and clinical profiling to enhance subgroup analysis and external validity.

Another important limitation is that the intervention was not limited strictly to surfing activity. The Operation Surf program includes several components that may contribute to observed outcomes, such as daily yoga and stretching, group reflection sessions, shared meals, informal peer support, and structured post-program contact through GroupMe chats and optional Zoom meetings. These elements continued beyond the in-person experience and may have influenced follow-up symptom scores. While surfing was the central activity and primary modality of engagement, it was embedded within a holistic, socially rich, and intentionally supportive environment. Because the study was not designed to isolate the effects of individual components, we cannot determine whether psychological or physiological improvements were attributable specifically to the surfing activity, the social environment, or their combination. Future studies may consider dismantling designs or multi-arm trials to assess the contribution of each intervention element.

This study intentionally evaluated the Operation Surf intervention as it is delivered in practice—a multi-faceted, community-based experience centered around surfing but inclusive of several complementary therapeutic components. The goal was to assess the holistic impact of the full program rather than isolate individual elements, aligning with the program’s design and participant experience.

Importantly, while the intervention may hold promise in terms of accessibility and community-based delivery, this study did not evaluate cost, stigma, or health policy outcomes. Therefore, we have refrained from making causal or evaluative claims about those domains. Future research should assess these dimensions directly, including economic evaluations, stigma assessments, and implementation science frameworks for policy integration.

In sum, this study advances the field of surf therapy by combining validated psychological instruments with objective physiological data to evaluate intervention outcomes among military veterans with PTSD. The results demonstrate statistically and clinically meaningful improvements in symptoms of anxiety, depression, and PTSD, alongside supportive trends in sleep and autonomic function. These outcomes suggest that multi-component, nature-based programs like Operation Surf hold real promise as scalable, community-based mental health interventions, and underscore the potential of nature-based interventions supported by digital health technologies.

Programs like Operation Surf may be particularly valuable because they engage veterans in non-clinical, peer-supported environments that have been described in prior research as more approachable and less stigmatizing than traditional care. While feasibility and methodological limitations exist, the integration of biometric tracking and participant-reported outcomes provides a robust foundation for future trials. As the field continues to evolve, incorporating flow theory, dose optimization, and biometric tracking alongside longitudinal follow up may yield deeper insights into therapeutic mechanisms and enhance the scientific foundation for complementary mental health approaches. As the field grows, future research should explore how such programs can complement traditional care, reduce access barriers, and foster long-term engagement in mental health recovery.

## Data Availability

The raw data supporting the conclusions of this article will be made available by the authors, without undue reservation.
